# Appointment—Dental Board

**Published:** 1898-07

**Authors:** 


### Appointment—Dental Board.

The term of office of the State Board of Dental Examiners of Ohio having expired in April last, the Governor of the State on the 30th of April appointed the following persons as members of that Board, to serve for three years; namely, Dr. A. F. Emminger of Columbus, Dr. L. P. Bethel of Kent, Dr. A.W. Price of Cleveland, Dr. O. N. Heise and Dr. M. H. Fletcher of Cincinnati. Soon after the appointment of these persons, they were called together and organized the Board by electing Dr. A. F. Emminger its President and Dr. L. P. Bethel Secretary.

This appointment meets the hearty approval of the profession of the State. The Governor will receive the sincere thanks of the dentists of the State for this excellent appointment. The Board proceeded at once with its legitimate work.

The profession in the State will doubtless in the near future receive a very marked impulse from the work of this Board.

